# Effect of T-Shape Shoulder Fillet on the Plastic Deformation Properties of SS400 and LYS160 Steel

**DOI:** 10.3390/ma13071528

**Published:** 2020-03-26

**Authors:** Chaofeng Zhang, Chen Shixi, Xuchuan Lin, Junhua Zhao, Quanlong Wang

**Affiliations:** 1 Institute of Engineering Mechanics, China Earthquake Administration; Key Laboratory of Earthquake Engineering and Engineering Vibration, 29 Xuefu Road, Nangang District, Harbin 150006, China; linxc03@gmail.com; 2 Jiangsu Key Laboratory of Advanced Food Manufacturing Equipment & Technology, Mechanical Engineering School of Jiangnan University, Lihu Road 1800, Binhu District, Wuxi 214100, China; chen635391143@163.com (C.S.); junhua.zhao@163.com (J.Z.); wangql@jiangnan.edu.cn (Q.W.)

**Keywords:** shoulder fillet, plastic deformation, strengthening effect, failure mode, fatigue performance

## Abstract

Shoulder fillets are widely used in the structural optimization design of metal dampers. However, the plastic deformation property of dampers affected by stress concentration, owing to different fillets, has not been explored in-depth. In this study, two typical metal damper materials with different plastic deformation, i.e., ordinary steel SS400 and low-yield-strength steel LYS160, were investigated. The strengthening effect of fillets under different loading is evaluated by comparing the mechanical properties of different fillet heights. Furthermore, the effect of the stress concentration caused by different fillet shapes, based on the failure mode of materials, is discussed. Subsequently, the fatigue degradation effect under the reciprocating shear loading is studied. Based on a series of studies on the deformation properties of fillets in different ductile materials, the basis for the structural optimization design under plastic deformation is provided.

## 1. Introduction

As an important energy-dissipating component, metal dampers are widely used in seismic engineering. It is crucial to optimize the design of metal dampers, to improve their energy-dissipation capacity, by fully utilizing the plastic deformation of metal materials.

Different types of dampers can be designed, using different deformation mechanisms of metal, such as shear [1], tension [2], bending, or their combination [3]. To utilize these dampers more effectively in energy dissipation, the core energy-dissipation structure is typically optimized. In the optimization design of a shear panel, a uniform stress distribution in the damper is desired. Hence, local weakening [4] or local strengthening [5] is generally performed. Local weakening is typically realized in shoulders with local penetration [6] or by holes with overall penetration [7]. Meanwhile, local strengthening is realized by adding stiffeners at weak positions [8]. 

In the case of dampers with tension and compression energy dissipation, the strength of the fixed part of the damper is generally significantly greater than that of the energy-dissipation core, to ensure that the latter can perform reliably. Accordingly, the thickness of the fixed part is greater than that of the energy-dissipation core [9,10], and a shoulder is set between them. From the optimization design of these dampers, it is clear that, regardless of the damper type, a reasonable design of shoulders is indispensable for an optimized design.

The fatigue performance of shoulders is generally evaluated by the stress concentration. Some studies focused on the stress concentration of shoulders under different loads, such as tension [11,12], bending [13], and shear [14,15]. At the same time, fillet height [16], fillet length [17], and fillet shape [18,19] of the shoulder are other research concerns. The results show that, when the fillet length is long enough, the stress concentration of the shoulder under different loads can be effectively improved. Accordingly, in order to reduce the influence of stress concentration, the length, height, and radius of the shoulder fillet are clearly specified in the national standard of material performance test [20].

**Figure 1:**
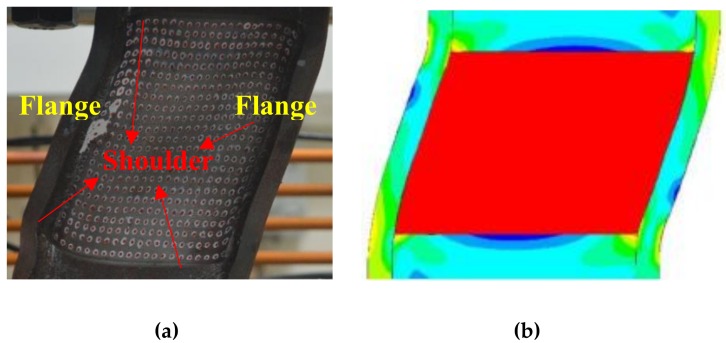
Shear panel damper (**a**) test result and (**b**) simulation result.

However, a large arc cannot be adopted in the thickness direction of a shear panel damper, owing to its thickness limitation. Therefore, a small arc fillet or a T-shape fillet [21] cannot be avoided. Through the rational design, the stress can be evenly distributed on the shear panel [22], as shown in Figure 1. The stress concentration under the traditional elastic deformation is usually represented as a narrow band of the maximum stress distributed at the fillet. The stress concentration of shear panel damper under plastic deformation is quite different with that of traditional shoulder under elastic deformation. 

Energy is typically dissipated by the plastic deformation of a damper instead of the elastic deformation. With the increase of loading cycle, the stress at the edge of shear panel decays rapidly when the plastic deformation reaches the critical value of fatigue [23]. Studies regarding the stress concentration of shoulder fillets under plastic deformation and its effect on the deformation properties of dampers are scarce. In view of the complexity of shoulder fillet damage under plastic fatigue loading, it is more convincing to analyze the deformation properties of shoulder fillet by experiment.

Compared with traditional ordinary steel, low-yield-strength steel is becoming more widely used in metal-damper design owing to its large plastic deformation. However, the effect of shoulder fillets in large plastic deformation on the structure’s performance has not been studied in-depth. Hence, the performance difference caused by shoulder fillets under different plastic deformation has not been compared and analyzed.

In this study, ordinary carbon steel and low-yield-strength steel were selected as test materials. The mechanical properties of ordinary carbon steel and low-yield-strength steel with different shoulder fillet shapes and heights under different loads were analyzed. The effect of T-shape shoulder fillet on the plastic deformation ability of structures fabricated by using these two different ductile materials is discussed in detail.

## 2. Research Plan 

### 2.1. Test Plan

**Figure 2:**
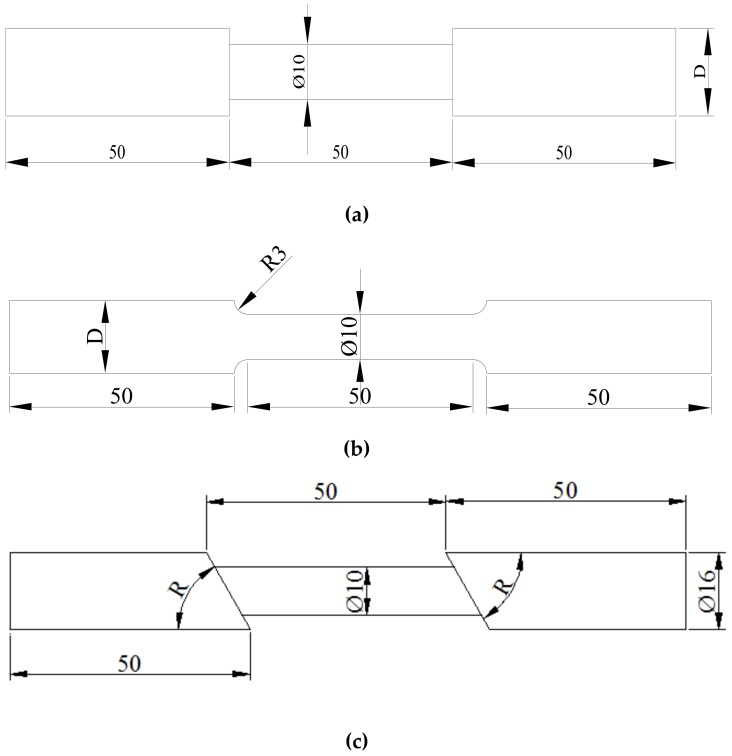
Specimen Diagram: (**a**) T; (**b**) ARC; and (**c**) OBLIQUE T.

Low-yield-strength steel (yield strength 160 MPa, LYS160) and ordinary carbon steel SS400 were adopted to design the specimens, as shown in Figure 2. The effective diameter of the specimen was 10 mm, and the length was 50 mm. To investigate the effect of fillet height under tension and shear, three different diameters (12/14/16 mm) of the clamped end were adopted. The diameter ratio of the clamped end to the specimen were 1.2, 1.4, and 1.6, respectively (Figure 2a,b).

Based on the three different fillet heights, the effect of fillet shape on structural deformation performance is further discussed. The properties of the standard specimen with a large arc transition can be obtained easily, according to previous studies [24]. Hence, they were not tested in this study. The fillet shape was designed as two types, i.e., T-shape with right angle and small arc, denoted as T and ARC, respectively. 

Flanges (Figure 1) are typically fixed on the left and right sides of the shear panel, to improve the stress. In the actual operating condition, the angle of these T-shape shoulders is no longer a constant value; it will change with the shear amplitude. Accordingly, three angles (R = 30°, 45°, and 60°) were designed in OBLIQUE T-shape shoulders (Figure 2c), to investigate the change in mechanical properties to clarify its effect on the plastic deformation capacity.

The research plan is shown in Table 1. Monotonic tension and shear tests were performed to investigate the effects of fillet height, fillet shape, and shoulder oblique angle. Subsequently, a constant amplitude fatigue test was performed for the T-shape shoulders. The low cycle fatigue damage degradation effects of different materials under reciprocating shear with the same fillet characteristics were compared and analyzed. The fatigue test plan is shown in Table 2.

### 2.2. Test Setup

**Figure 3:**
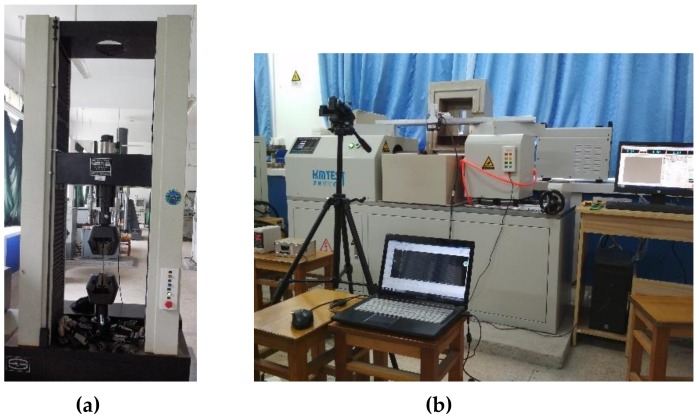
Test Setup: (**a**) Tension machine; (**b**) Torsion machine.

Tensile and shear tests were performed, using a universal material testing machine and a torsional fatigue testing machine with loading speeds of 1 mm/min and 180°/min, respectively (Figure 3). The force, displacement, torque, and twist angle of the specimens were measured directly by a computer. The experimental process was captured with a camera, and micro-section characteristics were observed through SEM (Scanning Electron Microscope).

## 3. Results and Discussion

### 3.1. Failure Mode

#### 3.1.1. Tension

**Figure 4:**
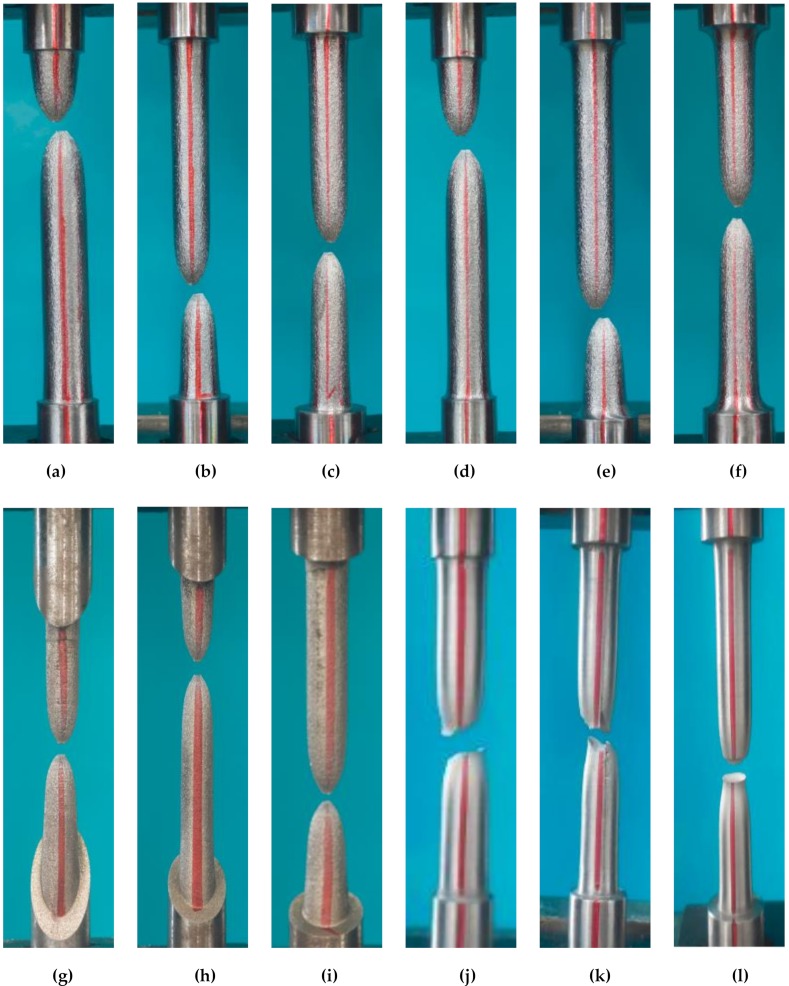
Failure mode of tension: (**a**) Ten1; (**b**) Ten2; (**c**) Ten3; (**d**) Ten4; (**e**) Ten5; (**f**) Ten6; (**g**) Ten7; (**h**) Ten8; (**i**)Ten9; (**j**) Ten10; (**k**) Ten11; and (**l**) Ten12.

The failure modes of specimens with different fillet heights under tensile loading are shown in Figure 4. No plastic deformation was observed in the clamped end of the specimens, even when the fillet height was the smallest (1 mm, Ten-1, Ten-4, and Ten-10). For LYS160 or SS400, a small fillet height can cause plastic deformation to be concentrated in the middle area. Under tensile loading, a smaller fillet height can yield a better step-strengthening effect.

All the specimens were necked and fractured in the middle parts. This implies that no stress concentration existed, or the stress concentration at the fillet was negligible under tensile loading. This phenomenon is primarily determined by the characteristics of tensile deformation, based on the following formula under tensile loading:

σ = F/A,(1)

where F is the tensile force; A is the section area, i.e., A = πd^2^/4; and d is the diameter of the specimen.

As the diameter of the clamped ends is larger than that of the middle part, the corresponding stiffness of the clamping part is greater than that of the middle part. Once the tensile loading is applied, the middle part will deform first. With the increase in loading, the middle diameter of the specimen decreases and the stiffness becomes smaller. The diameter and rigidity of the clamped part at both ends remained unchanged. Hence, the reduction in stiffness of the middle part caused the deformation to be concentrated in the middle part. The reinforcement effect of the clamped end can be realized when the diameter of the middle deformation area is slightly larger than that of the middle deformation area. Moreover, the stress concentration of the structure under tensile loading has little correlation with the fillet shape and angle of the shoulder.

#### 3.1.2. Torsion

**Figure 5:**
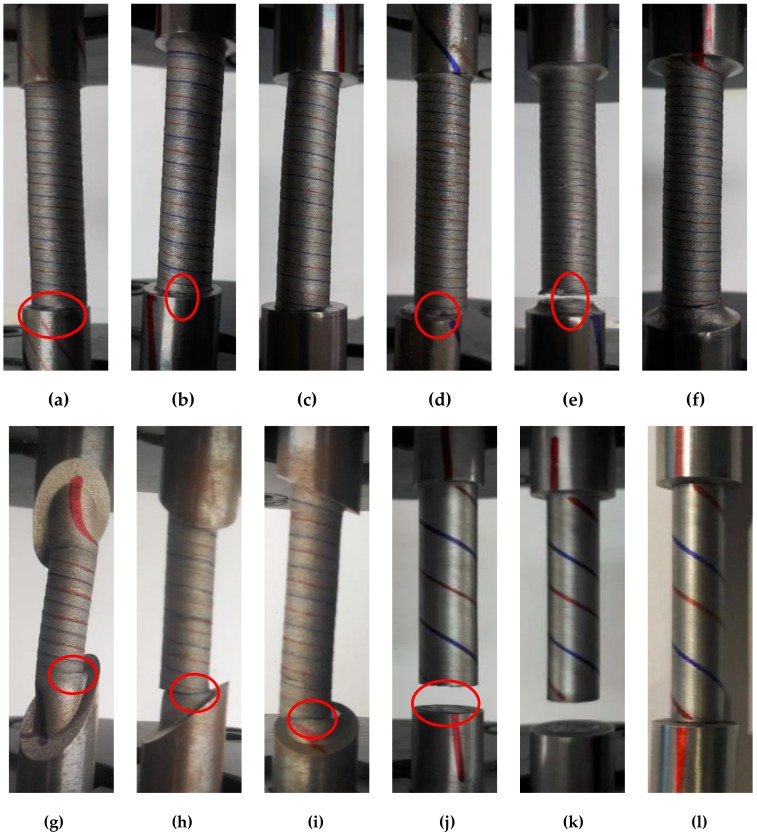
Failure mode of torsion: (**a**) Tor1; (**b**) Tor2; (**c**) Tor3; (**d**) Tor4; (**e**) Tor5; (**f**) Tor6; (**g**) Tor7; (**h**) Tor8; (**i**) Tor9; (**j**) Tor10; (**k**) Tor11; and (**l**) Tor12.

Red and blue lines were drawn on the opposite sides of the specimen, along the axial direction, to observe the torsion deformation of the specimen. The failure modes of the specimens under shear loading are shown in Figure 5. When the fillet height was small, plastic deformation was observed at two clamped ends. This indicates that, when the fillet height was 1 mm (D/d = 1.2), the clamping end could not produce a strengthening effect. When the fillet height was 2 mm (D/d = 1.4), the strengthening effect was demonstrated in specimen SS400. When the fillet height was 3 mm (D/d = 1.6), the strengthening effect was achieved in specimen LYS160.

Compared with the tensile test, the maximum shear stress of the torsion test is expressed as follows:

(2) $$\tau_{\max}=\frac{3}{4}\frac{T}{W_{t}},$$

where W_t_ = πd^3^/16; d is the diameter of the specimen; and T is the torque.

Without necking occurring in torsion, the shear area in the middle area of the specimen and the corresponding W_t_ remained unchanged. The torque increased with the deformation, owing to its stress-hardening effect. When the antitorque of the clamping end was lower than the loading torque, torsional deformation was produced in the clamping ends (Figure 5a,b,d,e). Therefore, the strengthening effect of the clamping end under torsional loading should be designed with consideration of the material’s hardening characteristics.

It was discovered that the fractures typically appeared at the fillet of the specimens under shear loading (Figure 5). High stress concentrations tend to be produced in the sharp transition part. When the clamping end shoulder was beveled, the stress concentration distributed at the shortest point of the middle specimen at the beveled edge. In fact, the effective length of the intermediate specimen was affected by the bevel angle. The stress concentration of the structure under shear loading was affected by the fillet height, fillet shape, and shoulder angle, simultaneously.

#### 3.1.3. Fracture Surface

**Figure 6:**
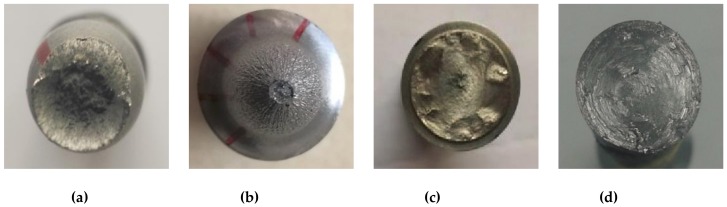
Failure morphology of transversal surface: (**a**) SS400 tension; (**b**) LYS160 tension; (**c**) SS400 torsion; and (**d**) LYS160 torsion.

The fracture transversal surfaces of specimens SS400 and LYS160 under different unidirectional loads are shown in Figure 6. Under tensile loading, necking was observed in both specimens SS400 and LYS160. The diameter of the necking section of specimen LYS160 was much smaller than that of specimen SS400 (Figure 6a,b), demonstrating a better plastic deformation capacity. Under shear loading, several peaks and troughs distributed at the fracture transversal surface of specimen SS400 (Figure 6c). The fracture transversal surface of LYS160 under shear loading exhibited a smooth plane (Figure 6d).

**Figure 7:**
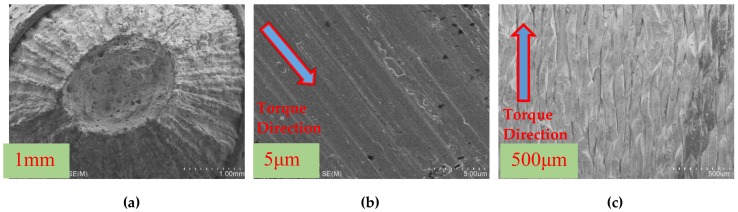
SEM of LYS160: (**a**)Tensile transversal surface; (**b**) Torsion transversal surface; and (**c**) Torsion cylindrical surface.

The microstructures of specimen LYS160 under tension and torsion are shown in Figure 7. Under tensile loading, many dimples appeared in the fracture transversal surface of specimen LYS160 (Figure 7a). Meanwhile, the surrounding surface of the specimen was distributed with grooves similar to those after plowing. Under shear loading, concentric circular slip lines were observed in the fracture transversal surface of specimen LYS160 (Figure 7b). Many parallel slip lines were also observed on the cylindrical surfaces of specimen LYS160 (Figure 7c). This demonstrated the good slip performance of LYS160 in both the axial and radial directions.

Seen from the fracture surface, the essence of failure mode of LYS160 and SS400 under tension was necking. The sole difference between them is the necking degree. The failure mode of LYS160 is totally different than that of SS400 under shear loading. LYS160 presents uniform interlayer slip deformation, with a story height of about 500 µm. A smooth slip surface can be formed between the layers, which is helpful for the stable energy dissipation of large plastic deformation.

#### 3.1.4. Cyclical Shear

**Figure 8:**
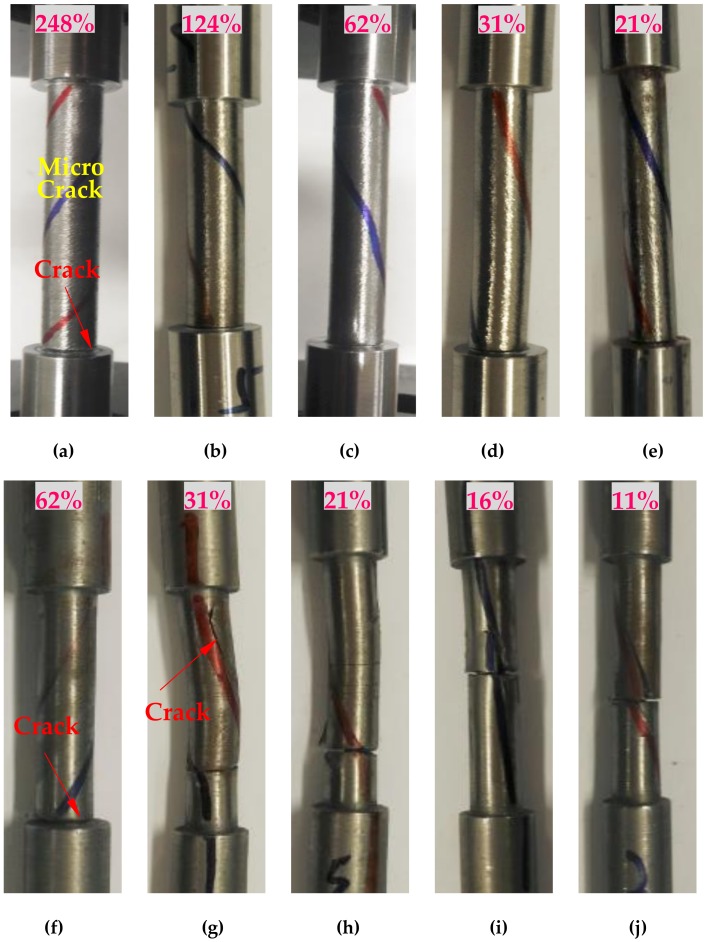
Failure mode of cyclical torsion: (**a**) CT1; (**b**) CT2; (**c**) CT3; (**d**) CT4; (**e**) CT5; (**f**) CT6; (**g**) CT7; (**h**) CT8; (**i**) CT9; and (**j**) CT10.

The failure mode of the T-shape shoulder specimen under the reciprocating torsion is shown in Figure 8. The former five figures are the failure modes of specimen LYS160, and the latter five figures are those of specimen SS400. When the fillet height was 3 mm (D/d = 1.6), plastic deformation was not observed at the clamping end. The strengthening effect of the clamp can be ensured under cyclical shear loading. The failure mode of specimen LYS160 under cyclical shear loading (Figure 8a–e) was the same as that under unidirectional shear loading, all of them fractured from the T-shape shoulder fillet.

The failure mode of CT6 (Figure 8f) was the same as that of the specimen under monotonic shear loading. Except for CT6, the failure mode of specimen SS400 under cyclic shear loading was consistent with that of the standard specimen under unidirectional loading. They almost cracked at the middle of the specimen (Figure 8g–j). The initial crack direction was consistent with the direction of the lines drawn on specimen SS400. The fatigue failure mode of specimen SS400 with a T-shape shoulder under cyclic shear loading depended on the loading amplitude. When the amplitude was large, the specimen with an abrupt shoulder fractured. When the amplitude was small, the failure mode of the specimen with a T-shape shoulder was the same as that of the standard specimen [24].

Similar to the monotonic shear loading, there were many parallel microcracks in the middle of the LYS160 specimen under the reciprocating shear loading, demonstrating a uniform interlayer slip. It suggests that the stress concentration is negligible in the middle part of the LYS160 specimens. Meanwhile, there was a sharp change of stress in the shoulder fillets, and the stress concentration was severe. Therefore, the corresponding failure mode is fillet crack. Under the reciprocating shear loading, the uniform deformation in the middle part of SS400 specimens cannot be obtained. The corresponding stress concentration is serious, and the failure mode of SS400 specimens was the fatigue fracture of middle part.

### 3.2. Stress–Strain Curve

#### 3.2.1. Monotonic Loading

**Figure 9:**
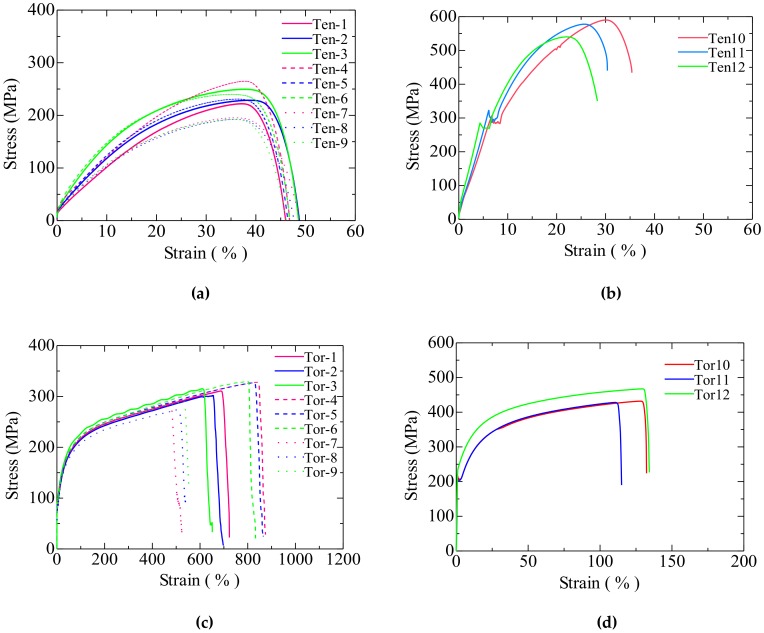
Stress–strain curves. (**a**) LYS160 Tension; (**b**) SS400 Tension; (**c**) LYS160 Torsion; and (**d**) SS400 Torsion.

The stress–strain curves of specimens with different shoulders are shown in Figure 9, and they did not differ vastly when tensile loading was applied (Figure 9a,b). The stress–strain curves of SS400 and LYS160 were nonlinear in the plastic range. The ductility of SS400 was poor, and it would break soon after necking, resulting in the sudden disappearance of the force. The ductility of LYS160 was pretty good. The force of LYS160 decreased gradually, from the maximum value to 0, after necking. The stress–strain curves of SS400 and LYS160 under torsion were approximate with linear in the plastic range. No matter the fillet form and material, the stress value dropped sharply once the fillet broke.

#### 3.2.2. Cyclical Torsional Loading 

**Figure 10:**
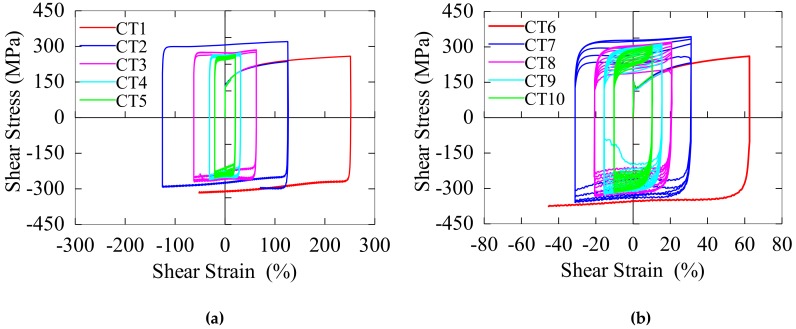
Hysteretic curve. (**a**) LYS160; (**b**) SS400.

The hysteresis curves of specimen LYS160 under the reciprocating torsional loading are shown in Figure 10a; and they exhibit a regular rectangular that can be simplified as a perfect elastic–plastic model. The hardening effect appeared in the second cycle, and there, no stress-hardening or stress-softening effect was observed from the second cycle. A significant decrease in force was observed once the specimen cracked. 

**Figure 11:**
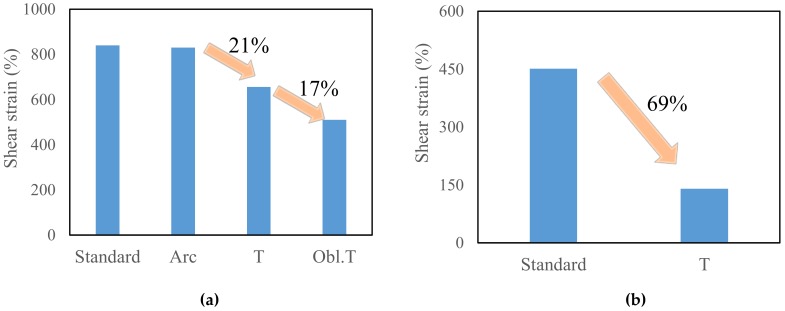
Deformation capacity: (**a**) LYS160; (**b**) SS400.

The hysteretic curves of specimen SS400 under the reciprocating torsional load are shown in Figure 10b; these curves were also approximately rectangular and could be simplified as a bilinear model. The hardening effect was observed in the second cycle. Subsequently, the continuous softening effect was observed from the third cycle. As shown in Figure 11, Specimen LYS160 exhibited a stable energy-dissipation capacity, whereas specimen SS400 exhibited stress degradation with the increase in cycle number.

### 3.3. Deformation Property

#### 3.3.1. Deformation Capacity under Monotonic Loading

When the shoulder was perpendicular to the loading direction, the effect on the plastic deformation capacity could be ignored. The maximum deformation capacity of LYS160 and SS400 was approximately 47% and 30%, respectively. Their ultimate strain values are approximate with that of the standard tensile specimen.

The deformation capacity of specimen LYS160 changed with the shoulder, under shear loading (Figure 11a). To compare with the standard torsional specimen with a large transition arc, the stress concentration can also be alleviated when the fillet radius is small. When the fillet radius was small (Tor-4, Tor-5, and Tor-6), the maximum shear strain was approximately 850%, which is similar to that of the standard torsional specimen [24]. 

When the shoulder fillet was T-shape, the stress concentration was distributed around the shoulder fillets of the specimen (Tor-1, Tor-2, and Tor-3). The deformation capacity of the specimen would be decreased by 21% if the T-shape shoulder fillet was adopted. When the T-shape shoulder was inclined, the stress concentration was concentrated at one point (Tor-7, Tor-8, and Tor-9). Compared with the T-shape shoulder specimens, the deformation capacity of the OBLIQUE T-shape shoulder specimen would be decreased by 17%. 

Compared with standard torsional specimen, the deformation capacity of SS400 decreased from 440% to 135% shear stain when the T-shape shoulder fillet was adopted. The reduction of deformation capacity was around 69% (Figure 11b). In the case of unidirectional shear loading, the material with poor plastic deformation capacity was more sensitive to fillet shape.

#### 3.3.2. Deformation Capacity under Cyclical Loading

**Figure 12:**
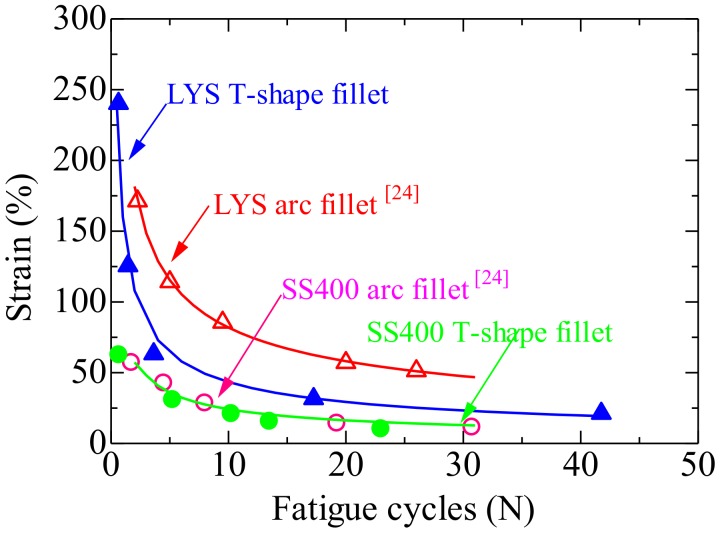
Fatigue performance.

The fatigue performances of the T-shape shoulder fillet and traditional standard specimens are shown in Figure 12. The red and blue lines are fatigue curves of the traditional standard and T-shape shoulder fillet specimens, respectively. As shown in Figure 12, the fatigue life of specimen LYS160 with a T-shape fillet was lower than that with an arc transition. When the number of cycles was 10, the strain amplitude of the traditional standard specimen was 82%, while that of the T-shape shoulder fillet specimen was 43.4%. The fatigue performance deteriorated by 47% in the large plastic deformation range when the T-shape fillet was adopted. 

The red and green circles in the figure are the fatigue cycles when the fillets of specimen SS400 are arc and T-shape, respectively. The green curve is the fatigue life curve. Although the maximum shear strain of sharp transition specimen SS400 was 69% lower than that of the standard specimen under unidirectional shear, the fatigue performance did not differ, owing to the small loading amplitude. The transition form of the fillet imposed little effect on the fatigue behavior of specimen SS400 under a small deformation.

Under reciprocating shear, the deformation in the middle of the LYS160 specimens was uniform, and the stress concentration was not significant. The stress concentration at the fillet caused the sharp decline of strain amplitude, and the advantage of large plastic deformation capacity of LYS160 could not be utilized. The fatigue performance of the specimens depends on the fatigue performance of the fillet, while it is less affected by the fatigue performance of the material. On the opposite, when the loading amplitude of SS400 is not more than 61% shear strain, the fatigue fracture occurs in the middle of the SS400 specimens. The fatigue performance of the specimens depends on the fatigue performance of the material, while it is not affected by the fillet. The difference of fatigue performance between different plastic materials is narrowed when the T-shape shoulder is adopted.

## 4. Conclusions

In this study, the deformation properties affected by different shoulder fillets were investigated by tensile and shear loading. The main conclusions are as follows:

(1) For LYS160 or SS400, its deformation capacity was not significantly affected by the transition form of fillets under tension loading.
(2) The shear deformation capacity was affected by the fillet shape, while it was insensitive to the fillet radius.
(3) Under reciprocating shear, the deformation capacity of large plastic material (LYS160) was affected by the T-shape fillet slightly, while the deformation capacity of small plastic material (SS400) was affected by the T-shape fillet greatly.
(4) Under repeating shear, stable interlayer deformation could be obtained in the middle part, and the deformation capacity of LYS160 specimens depended on the T-shape fillet. There was no stable deformation in the middle part, the deformation capacity of SS400 specimens was not affected by the T-shape fillet.
(5) When the fatigue cycle was 30, the corresponding amplitude of the LYS160 specimens with a T-shape fillet was 25% shear strain, which can meet most engineering applications.

## Figures and Tables

**Table 1:** Monotonic Loading Test.

Name	Material	Clamp Diameter (D) (mm)	Specimen Diameter (d) (mm)	Specimen Length (mm)	Fillet Height (mm)	Fillet Shape	Shoulder Angle (°)	Loading
Ten1	LYS160	12	10	50	1	T	90	Tension
Ten2		14			2			
Ten3		16			3			
Ten4		12			1	Arc	90	Tension
Ten5		14			2			
Ten6		16			3			
Ten7		16			3	Oblique T	30	Tension
Ten8							45	
Ten9							60	
Ten10	SS400	12			1	T	90	Tension
Ten11		14			2			
Ten12		16			3			
Tor1	LYS160	12			1	T	90	Torsion
Tor2		14			2			
Tor3		16			3			
Tor4		12			1	Arc	90	Torsion
Tor5		14			2			
Tor6		16			3			
Tor7		16			3	ObliqueT	30	Torsion
Tor8							45	
Tor9							60	
**Tor10**	**SS400**	**12**			**1**	T	**90**	**Torsion**
Tor11		14			2			
Tor12		16			3			

**Table 2:** Cyclical Torsion Tests.

Name	Material	Clamp Diameter (mm)	Specimen Diameter (mm)	Specimen Length (mm)	Fillet Shape	Loading Angle (°)	Shear Strain (%)	Loading
CT1	LYS160	16	10	50	T	1440	248	Cyclical Torsion
CT2						720	124	
CT3						360	62	
CT4						180	31	
CT5						120	21	
CT6	SS400					360	62	
CT7						180	31	
CT8						120	21	
CT9						90	16	
CT10						60	11	
